# The effect of meditative movement for glucose control in patients with type 2 diabetes: A protocol for systematic review and meta-analysis of controlled trails

**DOI:** 10.1097/MD.0000000000015639

**Published:** 2019-05-13

**Authors:** Tingwei Xia, Yue Yang, Weihong Li, Zhaohui Tang, Zongrun Li, Yongsong Guo

**Affiliations:** a Basic Medical College; b School of Clinical Medicine, Chengdu University of TCM, Chengdu, Sichuan Province, China.

**Keywords:** meditative movement, meta-analysis, qigong, systematic review, Tai Chi, type 2 diabetes, yoga

## Abstract

**Background:**

Type 2 diabetes is one of the most common and complex chronic disease. A lot of clinical researches have focused on meditative movement for type 2 diabetes. However, there is no systematic review and meta-analysis has been conducted. We aim to systematically review the effect of meditative movement on the type 2 diabetes care.

**Methods:**

The databases including PubMed, Cochrane Central Register of Controlled Trials (CENTRAL), Web of Science, Ovid LWW, EMBASE will be searched. Studies published from the time when the database establishment to December 2018 will be retrieved. RCTs study on meditative movement for glucose control in patients with type 2 diabetes will be included. The primary outcomes are HbA1c, fasting blood glucose, and postprandial blood glucose (PPBG). RevMan V.5.3 software will be used to perform the analyses.

**Results:**

This study will provide high-quality synthesis of effectiveness and safety of meditation movement for type 2 diabetes.

**Conclusion:**

This systematic review will provide high-quality evidence to judge whether the meditative movement is beneficial to glucose control in patients with type 2 diabetes.

**PROSPERO registration number:** CRD42019128495

## 1 Introduction

Type 2 diabetes is one of the most common and complex chronic disease. The pathogenesis is the body's resistance to insulin and the gradual decrease of insulin secretion by β-cells.^[1–3]^ Type 2 diabetes, also called “adult-onset diabetes” or “noninsulin-dependent diabetes,” accounts approximately for 90–95% of all diabetes.^[1]^ Typical symptoms of type 2 diabetes include thirst, frequent urination, fatigue, slow wound healing, repeated infections, and tingling or numbness of hands and feet.^[1]^ As the International Diabetes Federation reported, it is estimated that the number of people with diabetes will increase to 629 million by the end of 2045.^[4]^ Diabetes has brought heavy economic burden to individuals and society. It is estimated that there was $327 billion cost of diagnosed diabetes in 2017, including direct medical costs and reduced productivity. From 2012 to 2017, there is an increasing of 26% for the economic costs of diabetes.^[5]^ The diabetic complications have obvious specificity in the early stage and the later developmental stage of diabetes. Atherosclerotic cardiovascular diseases, such as coronary heart disease, cerebrovascular disease, or peripheral arterial disease, are considered to be the main causes of death in diabetic patients. An annual cost of $37.3 billion was estimated in cardiovascular-related spending associated with diabetes.^[6–11]^

Meditative movement, combining meditation, breathing, and relaxation, has been shown as a gentle exercise training.^[12]^ Tai chi, yoga, and qigong are regarded as the three typical forms of meditative movement. Tai chi and qigong are ancient Chinese exercise, while yoga as an exercise originated from ancient India. Yoga and Tai Chi are recommended by ADA for people with type 2 diabetes as a common exercise.^[13]^ At present, several systematic reviews or meta-analyses have shown that meditative movement is beneficial to chronic obstructive pulmonary disease, major depressive disorder, and sleep quality.^[14–18]^ However, there is no systematic review on the therapeutic effect of meditative movement on type 2 diabetes mellitus. The aim of this systematic review and meta-analysis is to evaluate the effectiveness of meditative movement for type 2 diabetes.

## 2 Methods

### 2.1 Data sources and selection strategy

We will perform a search in the following databases: PubMed, Cochrane Central Register of Controlled Trials (CENTRAL), Web of Science, Ovid LWW, and EMBASE. No language restrictions were imposed. Studies published from the time when the database establishment to December 2018 will be retrieved. An illustrative PubMed search strategy is as follows: “Tai Ji, Tai-ji, Tai Chi, Chi, Tai, Tai Ji Quan, Ji Quan, Tai, Quan, Tai Ji, Taiji, Taijiquan, T’ai Chi, Tai Chi Chuan” or “yoga” or “Qi Gong, Ch’i Kung” or “traditional Chinese exercise” or “meditative movement” and “Type 2 Diabetes Mellitus, Noninsulin-Dependent Diabetes Mellitus, Ketosis-Resistant Diabetes Mellitus, Non Insulin Dependent Diabetes Mellitus, Non-Insulin-Dependent Diabetes Mellitus, Stable Diabetes Mellitus, Type II Diabetes Mellitus, NIDDM Diabetes Mellitus, Noninsulin Dependent Diabetes Mellitus, Maturity-Onset Diabetes Mellitus, Maturity Onset Diabetes Mellitus, MODY Diabetes Mellitus, Slow-Onset Diabetes Mellitus, Maturity-Onset Diabetes, Maturity Onset Diabetes, Type 2 Diabetes, Adult-Onset Diabetes Mellitus.”

### 2.2 Inclusion criteria

Studies should meet the following inclusion criteria:

1. Participants: with a clear diagnosis of type 2 diabetes; without any serious complications.
2. Intervention: Tai Chi or Qigong or Tai Chi combined with Qigong or Yoga as intervention.
3. Control: any type of control group, including nonexercise in control groups or other active exercise control is acceptable.
4. Outcomes: primary outcomes are HbA1c, fasting blood glucose (FBG), and postprandial blood glucose (PPBG). Secondary outcomes are total cholesterol (TC), triglycerides (TG), high-density lipoprotein cholesterol (HDL-C), low-density lipoprotein cholesterol (LDL-C), and body mass index (BMI).
5. Study type: RCTs.

### 2.3 Data selection

**Figure 1:**
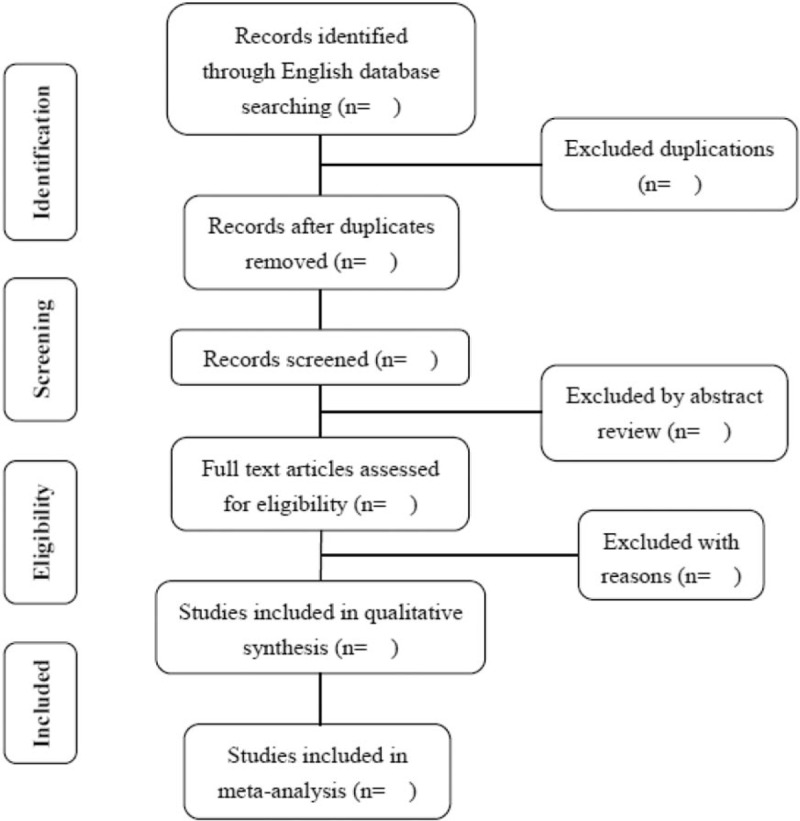
Flow diagram of study selection.

First, two independent investigators will search and screen the studies by finding duplications, excluding irrelevant titles and abstracts, and then selecting eligible studies by reviewing full texts. Abstracts that don’t meet the eligibility criteria are excluded, and those that don’t provide sufficient information about the inclusion criteria are further reviewed. Next, the same investigators will analyse the full texts, blinded to each other's review. Any disagreements between the reviewers will be resolved by consensus. The process of studies selection will be performed using the methods according to the PRISMA guidelines, presenting in the flow diagram (Fig. 1).^[19,20]^

### 2.4 Data extraction

Data extraction will be performed by two investigators independently. The data extraction is conducted by using pre-piloted, standardized forms, which contained basic information; methodological characteristics; participants’ demographic details; interventions details; outcomes; follow-up; and other. Finally, all differences will be resolved by consensus.

### 2.5 Methodological quality of assessment

Two investigators will independently assess the methodological quality of the included studies using RevMan 5.3.0, according to the Cochrane Handbook criteria for judging the ROB with the “Risk of bias” assessment tool.^[21]^ Seven domains should be evaluated, such as random sequence generation, allocation concealment, blinding of participants and investigators, the blindness of outcome assessments, incomplete outcome data, selective outcome reporting, and other biases. Based on the assessment, the quality of studies will be judged as low, unclear, or high bias.

### 2.6 Statistical analysis

RevMan 5.3.0, provided by Cochrane Collaboration, will be used to analyse the results of the studies. All outcomes are continuous variables, so we will express them as the st. mean ± standard deviation and then calculate the standardized mean difference (SMD) and obtain the two-sided *P*-value and 95% confidence interval (CI). We will use the complete case data as the analysis data. The χ^2^ test and *I*^2^ value are used to analyze heterogeneity. If *I*^2^ < 50%, the fixed-effect model is suitable to be employed to obtain synthesis results for studies with low heterogeneity. If *I*^2^ ≥ 50%, the random-effects model is adopted to assess the effect size for studies with significant heterogeneity.

We will perform subgroup analysis, as the meta-analysis of the primary outcomes show significant heterogeneity. Subgroup analysis will be on the basis of total sample size (>60 vs ≤60), duration (>3 months vs ≤3 months), control type (nonexercise vs other active exercise), type of meditative movement (Tai Chi/Qigong vs Yoga), region (Asia vs non-Asia), as well as a sensitivity analysis if necessary.

### 2.7 Publication bias

A funnel plot will be used to analyze whether there is a publication bias. The symmetrical funnel plots will indicate low risk, and asymmetrical funnel plots will indicate a high risk of publication bias.

## 3 Discussion

Exercise prescriptions have been widely used in the treatment of diseases. Meditation movement, as a special form of exercise, has a special effect on disease.^[22,23]^ Meditation movement is becoming more and more modeled, such as a biobehavioral model.^[18]^ In this way, it can treat respiratory diseases, tumors, and other diseases. Tai chi contains a series of slow, balanced, and concentrated movements accompanied by deep breathing.^[24]^ Qigong, including meditation, physical exercise, relaxation and breathing exercises, aims to control the body's vital energy (qi) along the energy channel (meridians). Yoga consists of pranayama, asanas, and meditation.

A lot of clinical researches have focused on this promising treatment for type 2 diabetes. However, no systematic review and meta-analysis has been found. Therefore, we intend to conduct a systematic review of meditation movement for type 2 diabetes in order to provide high-quality evidence of effectiveness and safety of meditation movement for type 2 diabetes, and provide reference for health policy makers and scientific researchers.

## Author contributions

**Conceptualization:** Tingwei Xia, Yue Yang, Weihong Li.

**Data curation:** Zongrun Li.

**Funding acquisition:** Weihong Li.

**Investigation:** Yue Yang.

**Methodology:** Tingwei Xia, Yue Yang.

**Project administration:** Tingwei Xia, Weihong Li.

**Software:** Yongsong Guo.

**Supervision:** Zhaohui Tang.

**Validation:** Weihong Li.

**Writing – original draft:** Tingwei Xia, Yue Yang.

**Writing – review & editing:** Tingwei Xia, Yue Yang.
