# The effect of meditative movement for glucose control in patients with type 2 diabetes: A protocol for systematic review and meta-analysis of controlled trails: Erratum

**DOI:** 10.1097/MD.0000000000037546

**Published:** 2024-05-03

In the article, “The effect of meditative movement for glucose control in patients with type 2 diabetes: A protocol for systematic review and meta-analysis of controlled trails”^1^, which appears in Volume 98, Issue 19 of *Medicine*, the final word of the title was misspelled and the correct title is “The effect of meditative movement for glucose control in patients with type 2 diabetes: A protocol for systematic review and meta-analysis of controlled trials.”
